# Breaking the Thumb Sucking Habit: When Compliance Is Essential

**DOI:** 10.1155/2016/6010615

**Published:** 2016-01-20

**Authors:** Orlando Tanaka, Wagner Oliveira, Melissa Galarza, Vanessa Aoki, Bruno Bertaiolli

**Affiliations:** Graduate Dentistry Program in Orthodontics, School of Health and Biosciences, Pontifícia Universidade Católica do Paraná, Curitiba, PR, Brazil

## Abstract

The anterior open bite (AOB) and posterior cross bite are the most frequent malocclusions associated with prolonged sucking habits. This clinical case illustrates and discusses the use of a Haas-type palatal expander for stopping a thumb sucking habit. The improvement in closing the open bite with discontinuation of the habit was observed. But with the return of the habit and lack of cooperation, the relapse of anterior open bite occurred. Therefore, different approaches are necessary. The need of a multidisciplinary approach, consent, and cooperation are keys to a good prognosis.

## 1. Introduction

The oral habits can interfere with the growth and normal development of the jaws, favoring the onset of malocclusion and changes in normal swallowing and speech patterns depending on factors such as duration, frequency, intensity, and facial pattern [1].

The open bite is most frequently seen in the anterior region, having deleterious habits as one of its main etiological factors. The opposition of the teeth deformation and the alveolar processes exhibit a configuration that is more or less a negative impression of the thumb or fingers, when they are placed in the mouth during sucking [1].

The AOB has several etiologic factors besides the finger sucking habit, such as incomplete eruption of the anterior teeth, predominantly mouth breathing habits, poor tongue posture, speech and tongue thrusting, pacifier habit, and a continuous abnormal swallowing pattern of the child.

Regardless of the finger used during suction, the duration, intensity, and frequency, combined with the patient's facial pattern, and the will and compliance to eliminate the habit should be evaluated [2]. Besides that, the early mixed dentition phase is appropriate for treating skeletal open bites [3]. The act of sucking his or her thumb seems to be very enjoyable and it can arise for various reasons, from a psychological problem to a discovery of a way to achieve pleasure. However, if the act remains for a long time, it becomes a damaging habit. Some malocclusions are corrected when the sucking habit stops, since the pattern is normal and the skeletal deformity is mild. But in many cases, this does not happen and orthodontic correction is needed [4], and even an orthognathic surgery [5].

In order to eliminate the finger sucking habit, there is no ready-made recipe. The use of provided adhesive plasters on the child's fingers, removable or fixed appliances, and even suggestion can be advocated [2, 6]. The preference is for braces, once it does not require collaboration, and the lack of compliance is part of the problem [1, 6, 7].

This case report aims to describe the normalization of the overbite when the thumb sucking habit is interrupted and the relapse if the habit returns and also to focus on the importance of the patient's cooperation to remove the habit and attention to a multidisciplinary approach.

## 2. Case Report

**Figure 1:**
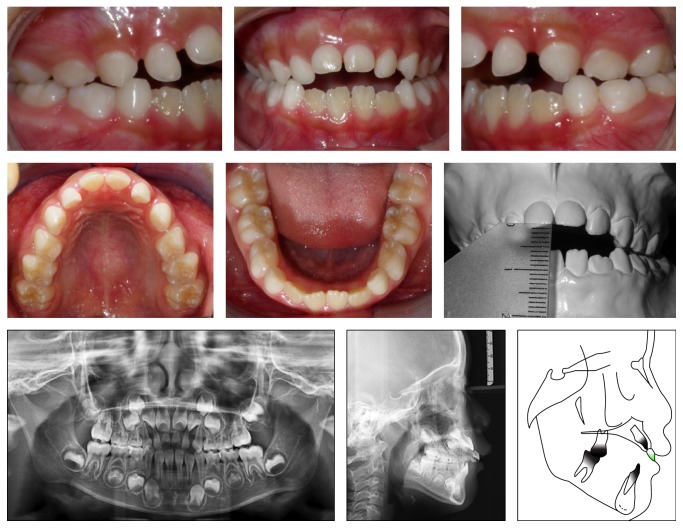
Pretreatment intraoral photographs, radiographs, and cephalometric tracing.

A 6-year-old and 8-month-old female patient, in mixed dentition, presented a Class II division 1 malocclusion, overjet of 9.0 mm, and AOB of 4.0 mm. Constricted maxillary arch, slight diastema between the maxillary incisors and between the lower central incisors, and a slight midline deviation to the left were also present (Figure 1).

The absence of second maxillary premolars was detected in the panoramic radiograph (Figure 1). The cephalometric measurements showed a good relationship between the maxillary and mandibular bases (ANB = 2°), proclined maxillary incisors (1NA = 32°), and uprighted mandibular incisors (1NB = 21°), a vertical growth trend greater than an anteroposterior (*Y*-axis = 61°) and a convex lower third of the face (*Z*-angle = 67°) (Table 1).

**Figure 2:**
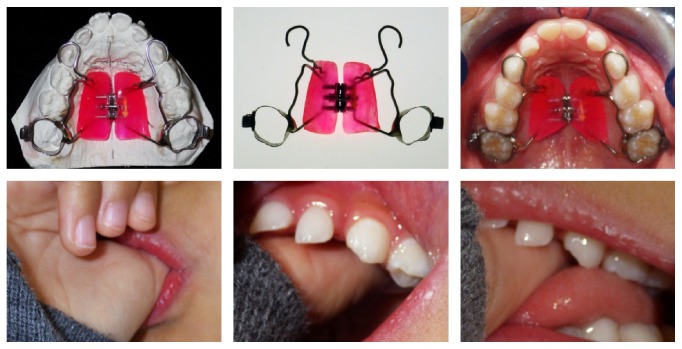
The expander before and after fixation. Thumb sucking position.

And the most important anamnestic information was regarding the presence of the thumb sucking habit of her right hand, and she reported that she was eager to stop sucking her thumb (Figure 2).

### 2.1. Treatment Objectives

Remove the thumb sucking habit, close the AOB, and allow the physiological eruption of the maxillary incisors by using a Haas-type palatal expander (Figure 2).

### 2.2. Treatment Alternatives

Among the interception alternatives for stopping the thumb sucking habit, the following were explained: RME with palatal crib, Palatal Nance button, helicoidal fixed palatal arch, maxillary removable appliance with crib, and motivational psychology and suggestion.

### 2.3. Treatment Progress

In the interception phase, it was explained to the patient and his parents the importance of their cooperation in the elimination of the finger sucking habit. It was also explained to them how the device and the trader could help stop the habit.

**Figure 3:**
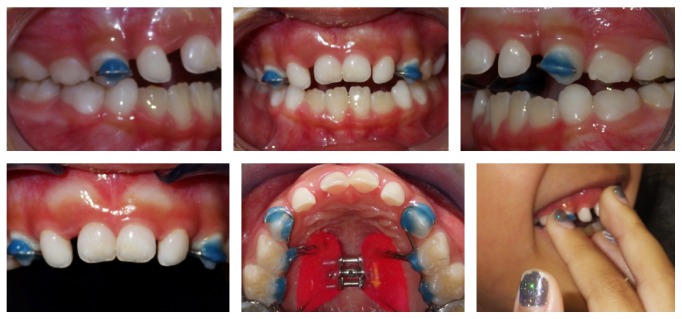
Progress after 3 weeks of palatal expansion. The appliance work as “reminder” and serve to curb the habit.

**Figure 4:**
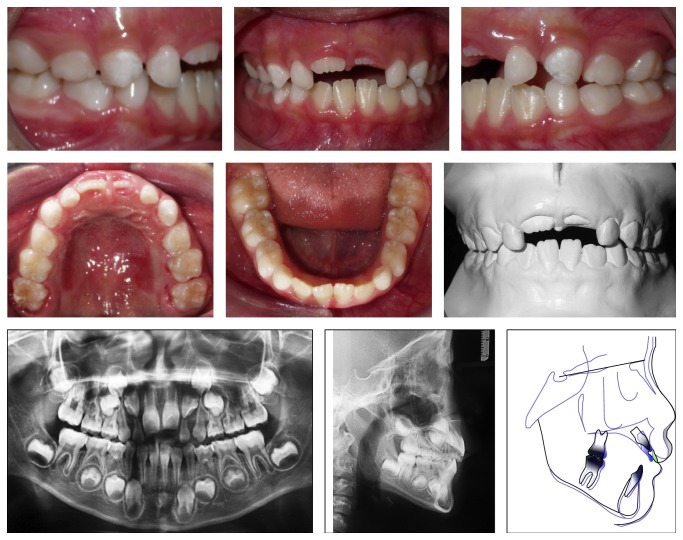
Postexpansion intraoral photographs, radiographs, and cephalometric superimposition. Black, initial; blue, final.

A fixed Haas-type appliance for RPE was planned and fixed in order to achieve palatal expansion and serve as a “reminder” to curb the habit and indirectly promote the closure of the anterior open bite (Figure 3). Orthodontic treatment was started with the fixation of the palatal device to the first molars and bonding on the palatal surfaces of the molars and deciduous canines (Figure 4).

### 2.4. Treatment Results

**Figure 5:**
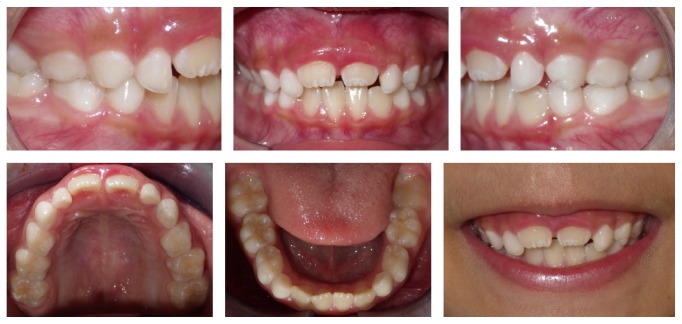
Nine months after removal of the palatal expander and no thumb sucking habit.

**Figure 6:**
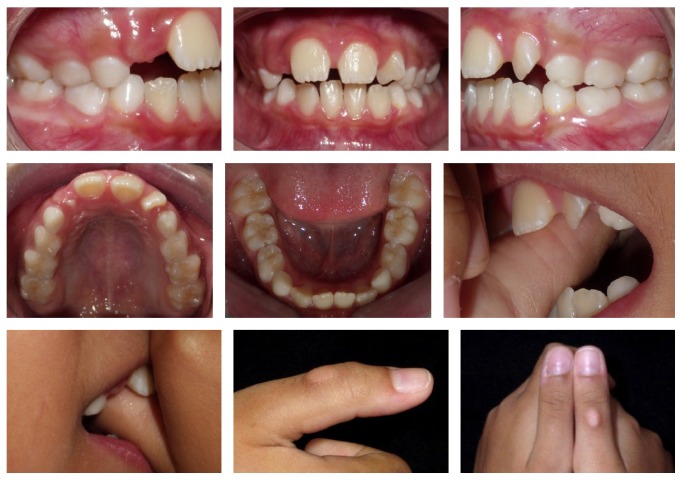
18 months after removal of palatal expander appliance. Relapse of the anterior open bite due to relapse of the thumb sucking habit. Callus on the right thumb observed.

During the first weeks of use of a palatal expander and in the subsequent months in the retention phase, the patient stopped her habit and the spontaneous correction of AOB was observed (Figure 5). The proposed objective of the RPE and stopping the thumb sucking habit were achieved with the functional reestablishment of occlusion. However, some months after the removal of the Haas-type palatal expander, the relapse of the habit was observed (Figure 6).

## 3. Discussion

Oral habits and AOB malocclusion have a high frequency in children. They hinder the normal development of dental and skeletal structures. As oral habits are risk factors for anterior open bite, the damaging habits most frequently associated are pacifier sucking, thumb sucking, and tongue thrust. Due to the correlation between the prevalence of anterior open bite and oral habits, prevention strategies incorporating psychological data related to children should be integrated into a national public health programme [8].

The AOB associated with deleterious habits can be successfully treated by means of interception at an early stage. However, the most important point in this therapeutic approach is the usual removal of the sucking habit [6, 9, 10]; otherwise, there will be a recurrence of malocclusion [11, 12].

The RPE is a common procedure in orthodontic mechanics and it is dedicated to expanding the maxillary arch; however, the preference for the Hyrax appliance has increased annually [13].

The etiologic factors most often associated with the development of an AOB are respiratory patterns [3], but age and growth factors also play an important role in AOB that is multifactorial, and there is an almost infinite variety to the dentoskeletal configuration and the magnitude of dysplasia associated with it [14].

Comforting behaviors, such as the use of finger or thumb sucking, are common in babies and young children but they tend to stop as children get older, under their own impetus or with support from parents and caretakers. However, if the habit continues whilst the permanent dentition is becoming established, it can contribute to, or cause, the development of a malocclusion. A diverse variety of approaches include advice, removal of the comforting object, fitting an orthodontic appliance to interfere with the habit, or behavior modification techniques. Some of these interventions are easier to apply than others and less disturbing for the child and their parents [15].

The maxillary removable or fixed appliance with crib is another alternative that not only prevents the suction of the finger or pacifier but also keeps the tongue in a more retruded position, preventing its interposition between the incisors during swallowing and speech. With the removal of the habit, there is a verticalization of the lower incisors and extrusion of the maxillary incisors closing the AOB [12] and similar results were observed in the present clinical case. The ideal age for the maxillary expansion is in mixed dentition, reducing the risk of damage and optimizing the procedure. As the patient progresses in skeletal age, the risk of damage to the supporting tissues increases particularly in growing patients.

The proposed results of maxillary expansion and stopping thumb sucking habit were fully achieved with the functional reestablishment of the occlusion. However, after the removal of the Haas-type appliance, it was observed the relapse of the thumb sucking habit with all its potential to harm the desired stability. Therefore, it is suggested that the desire to give up the habit must precede the presence of any type of appliance and that if after correction of the AOB the triggering factors of the usual reflexes are not completely eliminated.

There is low quality evidence that orthodontic appliances (palatal arch and palatal crib) and psychological interventions (including positive and negative reinforcement) are effective at improving sucking cessation in children [15]. When the habit is abandoned early, the malocclusion will usually revert without treatment. However, the absence of the habit is not a guarantee of having the malocclusion corrected [6] and the need for fixed appliances and complex biomechanics in the permanent dentition [16].

The fixed helicoid arch acts as a reminder and mechanical impediment of the habit and it causes discomfort for breaking the pleasure from the suction due to the volume of helicoids in the region where the pulp of the thumb should be [7]. During treatment, it should be clear to the patient, parents, or guardian that, for the successful removal of the finger sucking habit, it is not enough to use biomechanical orthodontic resources; the patient must be willing to stop the habit [6, 10].

A multidisciplinary approach should focus on efforts to build up the child's self-confidence and self-esteem.

## 4. Conclusion

The interception of the malocclusion at an early stage with the Haas-type fixed appliance was effective in RME and worked as an effective tool for stopping the thumb sucking habit and normalization of overjet and overbite. However, there was the recurrence of the habit with the relapse of the AOB, emphasizing the need of a multidisciplinary approach, consent, and cooperation as the keys to a good prognosis.

## Figures and Tables

**Table 1:** 

JF	Mean	Author	**6,9**	**7,3**
SNA	82	Steiner	**78**	**83**
SNB	80	Steiner	**76**	**77**
ANB	2	Steiner	**2**	**6**
Convex	0	Downs	**7**	**12**
*Y*-axis	60	Downs	**61**	**63**
Facial	88	Downs	**85**	**85**
SN-GoGn	32	Steiner	**39**	**38**
FMA	25	Tweed	**29**	**29**

IMPA	90	Tweed	**86**	**86**
1.NA	22	Steiner	**32**	**17**
1_NA	4	Steiner	**9**	**1**
1.NB	25	Steiner	**21**	**20**
1^−^NB	4	Steiner	**3**	**3**
Pog-NB		Holdaway	**0**	**1**
1 – 1	130	Downs	**125**	**138**
1^−^A-Po	1	Ricketts	**1**	−**2**

LS – S	0	Steiner	**5**	**3**
LI – S	0	Steiner	**1**	**1**
*Z*-angle	75	Merrifield	**67**	**69**
